# Is the concentration of C-reactive protein in bacteraemia associated with age?

**DOI:** 10.1186/1742-4933-5-8

**Published:** 2008-08-15

**Authors:** Astrid L Wester, Karl G Blaasaas, Torgeir Bruun Wyller

**Affiliations:** 1 Department of Bacteriology, Aker University Hospital, N-0514, Oslo, Norway; 2 Department of Microbiology, Ullevaal University Hospital, N-0407, Oslo, Norway; 3 Research Department, Aker University Hospital, N-0514, Oslo, Norway; 4 Faculty of Medicine, University of Oslo, Oslo, Norway; 5 Department of Geriatric Medicine, Ullevaal University Hospital, N-0407, Oslo, Norway

## Abstract

**Background:**

C-reactive protein (CRP) is an indicator of inflammation, and is often used in the diagnosis of bacterial infections. It is poorly known whether CRP in bacterial infection is age-dependent.

**Methods:**

Adult patients with a positive blood culture with *E. coli *or *S. pneumoniae *during 1994–2004 were included. CRP measured on the same date as the blood cultures were drawn (CRP1), 2–3 days (CRP2) and 4–7 days later (CRP3), were retrieved. The patients were divided into three age groups, < 65, 65–84, and ≥ 85, respectively. We studied three cut-off values for CRP and produced age-specific receiver operating characteristics (ROC) curves, using patients with acute coronary or cerebral infarction as controls.

**Results:**

890 patients and 421 controls were available. There was a statistically significant negative correlation between age and CRP1 – 0.072 (p = 0.032). The median CRP1 and CRP2 were significantly higher in the youngest age group. The area under the ROC-curve for the youngest age group was significantly greater than that of the two other age groups, but we found no statistically significant differences in sensitivity related to age. The diagnostic sensitivity of CRP was better for *S. pneumoniae *than for *E. coli*, 92.6% vs. 88.0% (p = 0.046) for a cut-off value of 40 mg/L, and 82.4% vs. 61.5% (p =< 0.01) for a cut-off value of 120 mg/L.

**Conclusion:**

CRP is better in identifying infection with *S. pneumoniae *than with *E. coli*. We found a weakening of the CRP-response with age, but this is hardly of clinical significance.

## Background

C-reactive protein (CRP) is the prototypical acute phase protein in humans [1,2]. It is widely used as a marker of infection, and also to aid the differentiation between a bacterial and a viral origin. Many geriatricians have the clinical impression that the CRP response to serious invasive bacterial infections may be delayed in frail old patients. This view is not supported in the literature [1], but studies in this field are rather scanty. Some relatively small studies exist [3-5], but were not designed to compare the diagnostic sensitivity for bacterial infections in different ages, and do not give information on the proportion of false-negative tests.

CRP is mainly produced by the liver [6]. The synthetic function of the liver may be reduced due to physiological aging, possibly due to both decreases in liver volume, liver blood flow and perfusion, and to cellular changes [7]. The main stimulator of CRP synthesis in the liver is interleukin 6 (IL-6), which is associated with diseases of old age such as multiple myelomas, chronic lymphatic leukaemia and renal cancer [8], and is higher in older than in younger subjects [9]. In mice the regulation of IL-6 synthesis is dependent of dehydroepiandrosterone, a hormone that declines with age, and the dysregulation is reversed by replacement of the hormone [10]. Studies have found a delayed IL-6 response in infection in the old [11], as well as impaired production of proinflammatory cytokines [12]. Thus, a diminished or delayed CRP response to bacterial infections in the old may be biologically plausible. The issue is clinically important, as the atypical clinical symptoms and signs of infection in geriatric patients make a correct diagnosis more dependent on a rational use of supplementary tests.

Hogarth et al suggest a cut-off value of CRP at 40 mg/L to be sufficient in elderly patients with infection [13], whereas one study has recommended that the cut-off value indicating sepsis, irrespective of age, should be 80 mg/L [14]. Even higher cut-off levels are also commonly used in clinical decisions.

*E. coli *is the most frequent Gram-negative species in bloodstream infections [15], whereas *S. pneumoniae *is the leading cause of community-acquired pneumonia and a significant cause of bacteraemia and meningitis [16]. Bacteraemia with either of these species will in almost every instance represent a clinically important invasive infection in need of antibacterial treatment. A "normal" CRP-concentration in these conditions can therefore be considered a false negative test result if CRP should be regarded as an indicator of bacterial infection.

The main aim of this study was to assess the diagnostic sensitivity of a raised CRP-concentration for bacteraemia with either *E. coli *or *S. pneumoniae *in different age groups. We also studied the impact of different cut-off points for the CRP-concentration as well as differences in the time course of the CRP-response in relation to age.

## Methods

### Setting and selection of patients

Aker University Hospital is a 350-bed hospital in Oslo, Norway, serving a population of 500 000 for urology and abdominal vascular surgery, and 180 000 for internal medicine, general surgery and psychiatry. Until the late 1990's, the hospital served the latter population in gynaecology, obstetrics and paediatrics as well.

All adult (≥ 16 years) patients with culture-verified bacteraemia of *E. coli *or *S. pneumoniae *during the period 1994–2004 were retrieved from the database of the hospital's bacteriological laboratory. Patients having more than one episode of bacteraemia were registered only once. For analytical purposes, the patients were divided into three age groups, < 65, 65–84, and ≥ 85, respectively. To be able to evaluate the need for age differentiated cut points for CRP, we selected patients admitted to the Medical department with ICD 10 diagnoses corresponding to either acute myocardial or acute cerebral infarction during 2003 to serve as controls. Patients with any ICD10 diagnoses indicating severe infection or a positive blood culture with a significant bacterium were, however, not used as controls.

### Microbiological methods

Blood was cultured by the Bactec 9240 system (Becton & Dickinson, Sparks, MD, USA). Further identification of the positive blood cultures followed standard procedures of the laboratory. Growth of possible *S. pneumoniae *was confirmed by a typical phenotype, sensitivity to optochin and by an agglutination test (Slidex pneumo-Kit, BioMériux, Lyon, France). *E. coli *was identified by a three tube method for identification of Gram-negative rods [17], or by the api20E system (BioMériux).

### CRP analysis

From the databases of the clinical chemistry laboratory, we retrieved the CRP concentration in blood drawn on the same date as the blood culture, if applicable (CRP1). When available, we also retrieved one CRP value measured on the second or third day of the hospital stay (CRP2), and one retrieved between day four and day seven (CRP3). All the CRP-measurements were performed on automated clinical chemistry analyzers (Roche Diagnostics, Mannheim, Germany): first on Hitachi 717, then Hitachi 917 and then on Modular. The assay vendor in the actual period was Roche diagnostics. The assay is based on an immunoturbidometric principle, where CRP is agglutinated with anti-CRP. In 2004 the assay was changed to a latex-particle-bound-anti-CRP reagent, which gave an enhanced agglutination signal with CRP. No significant shift in results could be detected at the time of change, comparing the old and new methods in selected clinical specimens. The analytic stability over the time period was documented by stable values of daily long-time quality control specimens. The reference range was unchanged during the period.

In order to test for any shift in the analyses over time in the present material, we estimated the Pearson correlation coefficient between time of testing and CRP concentration in a subsample of 681 patients. The correlation coefficient was non-significant at 0.060 (p = 0.12). When sub grouping the material according to time of change of analyzers and type of reagent kits, no statistically significant differences were found neither in mean ranks (p = 0.065) nor in medians (p = 0.398).

### Statistical methods

Since CRP-data and age were not normally distributed, data are presented as medians and interquartile ranges (IQR), and Mann-Whitney U tests were used to compare the ranks of age in the bacteraemic patient group versus the control group, and to compare the ranks of CRP1, CRP2 and CRP3 in the three different bacteraemic patient age groups. The Spearman rank correlation was used to examine a possible linear association between age and CRP1. The medians and IQRs of CRP1, CRP2 and CRP3 were depicted graphically. Contingency tables were used to calculate the sensitivities of CRP1 according to age group, bacteria, gender, and cut-off value (40, 80 and 120 mg/l, respectively). The confidence intervals of the sensitivities were manually calculated, and Chi-squared tests were used to compare the observed proportions. A 10% difference in sensitivity was considered as clinically relevant. A power analysis indicated that 294 patients in each age group would be required to detect such a difference with a power of 80% and a 5% significance level. Receiver operating characteristics (ROC) curves for the three age groups were constructed, and area under the curves (AUC) was calculated. Z-tests were used to assess whether the AUCs were statistically significantly different.

All tests were computed using SPSS -15.0 (SPSS Inc. Chicago, IL, USA), or by hand. P-values less than 0.05 were regarded as a sign of statistical significance. No attempts were done to adjust for multiple comparisons.

## Ethical considerations

The study was approved by the Regional Committee for Ethics in Medical Research and by the Norwegian Data Inspectorate, which gave permission to carry out the study without the patients' consent.

## Results

1150 patients had a positive blood culture with either *E. coli *or *S. pneumoniae *during the study period. Of these, 260 had no registered CRP measurement on the same day as the blood culture, reducing the number of eligible patients to 890. Of these, 604 (68%) had had their blood culture and CRP drawn on the day of hospital admittance. 634 (71%) of the patients had *E. coli *whereas the remaining 256 had *S. pneumoniae*. Median age of bacteraemic patients was 75 years (IQR 58–82). 300 (34%) patients were < 65 years, 443 (49%) were 65–84 years, and 147 (17%) were ≥ 85 years. The percentages of *E. coli *in the three age groups were 61%, 77% and 76%, respectively (p =< 0.01), and the percentages of females were 58%, 50% and 71% (p =< 0.01), respectively. 421 control patients were eligible. Their median age was 77 years (IQR 62–83), which was significantly higher than in the bacteraemic group (p = 0.005). 120 (28%) of the controls were < 65 years, 223 (52%) were 65–84 years, and 84 (20%) were ≥ 85 years.

**Figure 1:**
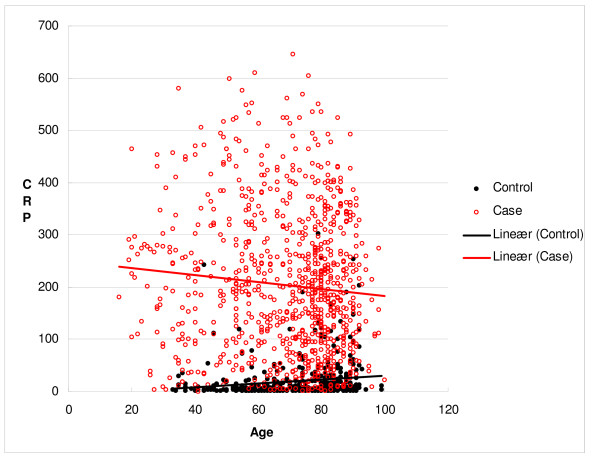
Scatter plot of the relation between age and CRP concentration on the day of a blood culture positive for *S. pneumoniae *or *E. coli *(cases), and for the controls.

The median concentration of CRP on the same day as the positive blood culture (CRP1) was 188 mg/L (IQR 97–288 mg/L). For the control group taken together the median CRP concentration at admittance was 6 mg/L (IQR 2–19 mg/L, range 1–303 mg/L), 5 mg/L (IQR 2–12 mg/L) for those with cerebral infarction and 7 mg/L (IQR 2–24 mg/L) for those with myocardial infarction. The relation between CRP1 and age with trendlines is shown in figure 1. The Spearman correlation coefficient was – 0.072 (p = 0.032) and 0.146 (p = 0.003) among the cases and the controls, respectively. For bacteraemic patients the median CRP1 was 209.5 (IQR 110–320), 175 (IQR 83–271) and 176 mg/L (IQR 103–278), in the youngest, median and oldest age group, respectively. Corresponding results among the controls were 4 (IQR 2 – 11), 6 (IQR 2 – 20) and 11 mg/L (IQR 3 – 43).

**Figure 2:**
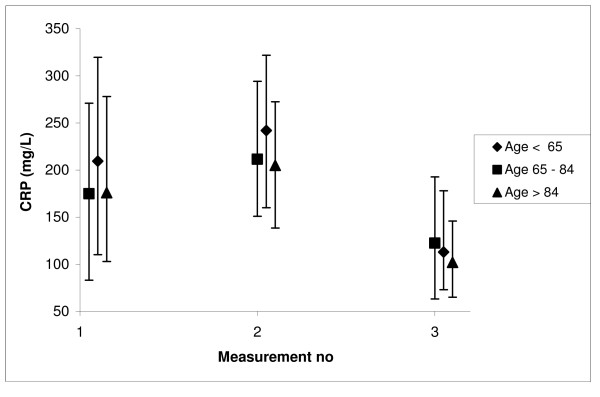
Median CRP1 (on the day of the positive blood culture), CRP2 (2–3 days later), and CRP3 (4–7 days later) in different age groups. The vertical bars indicate the interquartile range.

For bacteraemic patients, the medians and IQRs of CRP1, CRP2 and CRP3 in the three age groups are shown in Figure 2. The youngest group had a significantly higher CRP1 level than the intermediate group (p = 0.003). For CRP2 both the difference between the youngest and the intermediate group (p = 0.04) and the difference between the youngest and the oldest group (p = 0.026), were statistically significant. There were no significant differences for CRP3.

The sensitivity of CRP in detection of bacteraemia according to age group, bacterial species and gender at different cut-off values is shown in table 1. At all cut-off points, the sensitivity is significantly better for *S. pneumoniae *than for *E. coli*, but we were not able to identify any differences in diagnostic sensitivity regarding age groups.

**Table 1:** Sensitivity of a "high" concentration of C-reactive protein (CRP) as indicator of bacteraemia by gender, species, and age

		**Sex**		**Species**		**Age group**		
		Female	Male	*S. pneumoniae*	*E. coli*	16–64	65–84	≥ 85
**Cut-off (CRP) 40 mg/L**	Sensitivity (%)	90.2	88.2	92.6	88.0	90.4	88.5	90.5
	95% CI	87.6 – 92.8	85.0 – 91.4	89.4 – 95.8	85.5 – 90.5	86.6 – 93.4	85.5 – 91.5	85.8 – 95.2
	p-value	0.339		**0.046**		0.714		
								
**Cut-off (CRP) 80 mg/L**	Sensitivity (%)	81.4	75.6	89.1	74.8	82.0	75.6	82.3
	95% CI	78.0 – 84.8	71.3 – 79.9	84.8 – 93.4	71.4 – 78.2	77.7 – 86.3	71.6 – 79.6	76.1 – 88.5
	p-value	**0.037**		**< 0.01**		0.060		
								
**Cut-off (CRP) 120 mg/L**	Sensitivity (%)	69.7	64.6	82.4	61.5	71.7	64.8	67.3
	95% CI	65.7 – 73.7	59.9 – 69.3	77.7 – 87.1	57.7 – 65.3	66.7 – 76.8	60.4 – 69.2	59.7 – 74.9
	p-value	0.106		**< 0.01**		0.145		

CI = confidence interval, p-values for between-group comparison of sensitivity (chi-square test)

**Figure 3:**
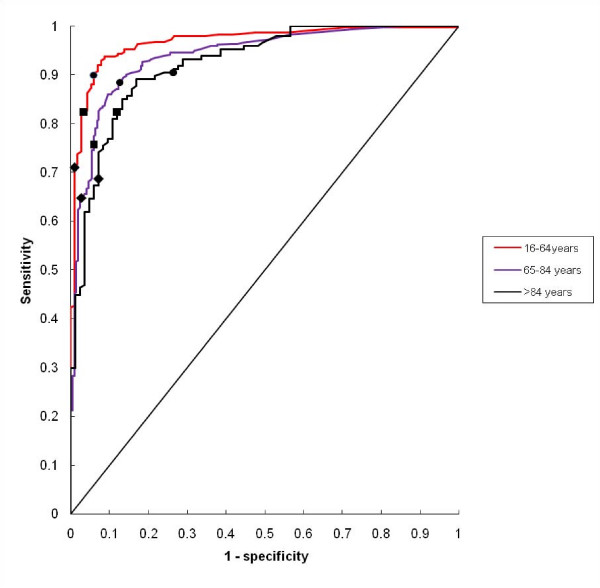
**Receiver operating characteristics (ROC) curves for the relationship between initial CRP value and bacteraemia in different age groups**. The sensitivity and the corresponding 1-specificity for the cut-points 40, 80 and 120 mg/L are indicated by circles, squares and diamonds, respectively.

The ROC-curves for the different age groups are shown in figure 3. The AUCs are 0.968 (95% confidence interval (CI) 0.952–0.985), 0.940 (95% CI 0.922–0.958) and 0.923 (95% CI 0.888–0.958) for the age groups 16–64 years, 65–84 years, and ≥ 85 years, respectively. The AUC for the youngest age group was significantly greater than that for the intermediate as well as the oldest age group (z-test, p < 0.05 for both comparisons).

## Discussion

We were not able to confirm the hypothesis that the CRP-response in bacteraemia is age-dependent to any clinically important degree. There was a slight, though statistically significant negative correlation between age and initial CRP value as well as a higher area under the ROC curve for the youngest patients, but this did not translate into a clinically meaningful difference in sensitivity for any relevant cut-off value for CRP. The study may have been underpowered for a comparison of sensitivity, but when the age groups were collapsed into broader classes (results not shown), thus improving the power, the negative results regarding sensitivity persisted.

Accordingly, this study supports the use of CRP as an indicator of possible infection in the elderly with the same cut-off values as in younger adults. This result should, however, be interpreted with several reservations.

First, we cannot completely rule out selection bias, since we do not know if the blood cultures in some instances were drawn after the results of the CRP-tests were known, thus increasing the possibility that some cases of bacteraemia remained undiagnosed in cases with low CRP. Moreover, fever is a very common indication for having a blood culture drawn, but is less frequent in elderly patients with infection [18]. Both factors could bias the results towards higher CRP levels in the elderly patients, and this trend may be strengthened by the fact that elderly patients more often have non-infectious comorbid conditions characterized by high CRP concentrations.

Second, we lack information on the duration of symptoms prior to CRP testing. A substantial proportion of the patients had their CRP1 drawn at admission. If there exists a systematically increased pre-hospital delay among the oldest, a slower CRP response in this group could be masked.

Third, we have insufficient clinical information as to include the potential confounding factors of comorbidity, disability or frailty upon the CRP response, an association that has been suggested by others [19,20]. Neither do we have sufficient information on the clinical severity of the infection.

It should be noted that our results are valid only for bacteraemia with *E. coli *or *S. pneumoniae*. Our study was not designed to assess the sensitivity of the CRP-response for severe bacterial infections that does not result in a positive blood culture for one of these bacteria. Thus, one should be careful in generalising our results to different clinical settings. Furthermore, the choice of control group can be criticized as both cerebral and coronary infarction may lead to elevated CRP. However, the choice was based on clinical grounds as these two conditions represent the main alternative diagnoses in the emergency room, especially when dealing with elderly patients.

The small relative difference between the age strata persisted at day 2–3, but disappeared at day 4–7 (Figure 2). This weighs against the hypothesis that the CRP response among the oldest is delayed, as in this instance we would have expected the progress from the first to the second assessment to differ according to age. It also weighs against the hypothesis of a prolonged inflammatory response in human old age [11], as is seen in aged mice [21]. There may, however, have been a selection bias in the ordering of a second and a third measurement. We have no information on the reasons why these repeated measurements were ordered.

Thus, taking these important reservations into account, there is still a real possibility that there may exist a clinically relevant age related difference in CRP response in systemic infection, at least in invasive bacterial infections not causing a positive blood culture. What factors are in favour of this hypothesis?

In ageing research it is widely accepted that ageing is characterized by a systemic, low-grade inflammatory state [22]. It is also acknowledged that this subclinical proinflammatory status, interacting with genetic background (evolutionary selection of proinflammatory genes beneficial in early age), may trigger the onset of frailty and age-associated diseases [22-24]. Innate immunity is viewed as being mainly well preserved, but immune changes seen in old age also affect innate immunity [24]. Studies on possible age-related changes in proinflammatory cytokines (and thus the CRP response) in endotoxiemia have yielded diverging results [25-28]. A recent study on gene expression in endotoxin-stimulated macrophages from young and old mice showed reduced expression of proinflammatory cytokines as well as Toll like receptor signalling pathways with increasing age [29]. Another study showed that the gene expression of proinflammatory cytokines was significantly lower in aged mice than in young at 24 hours after stimulation with endotoxin, but higher at 72 hours [21]. Thus, although still controversial, experimental studies do open up for a weaker proinflammatory response and thus a limited production of CRP.

There seems to be a convincing and clinically important difference in the sensitivity of CRP between patients infected with *E. coli *and *S. pneumoniae*. This finding accords well with the results of other studies. The CRP concentration is associated with mortality and organ failure [30], and it is well known that *S. pneumonia *consistently causes serious infections, while the range of severity is much broader for infections with *E. coli*. The finding of a gender difference in sensitivity in one particular age stratum, on the other hand, seems not biologically plausible, and we suspect this association to be spurious.

## Conclusion

We conclude that there exists a statistically significant association between age and CRP in invasive bacterial infections. However, this association may not be of a magnitude that makes it clinically important for CRP as a diagnostic tool in bacterial infections associated with a positive blood culture. Further studies are needed to decide whether markers of the aging process such as comorbidity and frailty influence the diagnostic value of CRP as a marker of infection, as well as whether the diagnostic performance of CRP is affected by severity of infection.

## Competing interests

This study was financed by Aker university hospital and by Ullevaal university hospital. The authors have no competing interests.

## Authors' contributions

ALW participated in all parts of the study, both in the design of the study, data collection, statistical analysis and interpretation, and in the writing of the manuscript. KGB participated in performing the statistical analysis, the interpretation of the data and in the writing of the manuscript. TBW participated in the design of the study, the interpretation of the statistical analysis and in the writing of the manuscript, including revising it critically for important intellectual content.

All authors have read and approved the final manuscript.
